# Supplementary material for: Overexpression of Aurora Kinase B Is Correlated with Diagnosis and Poor Prognosis in Hepatocellular Carcinoma
Source: Int J Mol Sci. 2024 Feb 12;25(4):2199. doi: 10.3390/ijms25042199 (PMC10889672; doi:10.3390/ijms25042199)
Supplement: Supplementary file 1 [file ijms-25-02199-s001.zip › ijms-2735583-supplementary.pdf]

Supplementary Table S1 Information on immunohistochemical samples for AURKB.

Sample	Tissue Type	ID	Age	Gender	Staining	Intensity	Quantity
1	Normal	1846	32	female	Not detected	Negative	None
2	Normal	1720	67	male	Not detected	Negative	None
3	Normal	1899	29	female	Not detected	Negative	None
4	HCC	2280	80	male	Low	Moderate	<25%
5	HCC	983	53	female	High	Strong	75%-25%
6	HCC	82	66	female	Not detected	Negative	None
7	HCC	2177	78	female	Not detected	Weak	<25%
8	HCC	1163	55	male	Not detected	Weak	<25%
9	HCC	2556	72	male	Not detected	Negative	None
10	CHOL	2279	73	female	High	Strong	75%-25%
11	CHOL	937	65	female	High	Strong	75%-25%
12	CHOL	2578	50	male	Medium	Moderate	75%-25%
13	CHOL	2399	52	female	Not detected	Negative	None
14	CHOL	877	61	female	Low	Moderate	<25%
15	CHOL	952	68	female	Low	Moderate	<25%

HCC, Hepatocellular carcinoma; CHOL, Cholangiocarinoma

Supplementary Table S2 Reports of AURKB overexpression in human neoplasms.

Tumour Type	References
Hepatocellular cancer	[29]
Non-small cell lung cancer	[64,65]
gastric cancer	[56,66]
neuroblastomas	[67]
Prostate cancer	[68]
Testicular cancer	[69]
Colorectal carcinoma	[70,71]
Breast cancer	[72]
AML	[73]
chondrosarcoma	[54]
epithelial ovarian cancer	[74]
multiple myeloma	[55]
Intrahepatic cholangiocarcinoma	[75]
